# Clinical aspects of human rabies in the state of Ceará, Brazil: an overview of 63 cases

**DOI:** 10.1590/0037-8682-0104-2021

**Published:** 2021-07-23

**Authors:** Naylê Francelino Holanda Duarte, Roberto da Justa Pires, Victoria Forte Viana, Levi Ximenes Feijão, Carlos Henrique Alencar, Jorg Heukelbach

**Affiliations:** 1 Universidade Federal do Ceará, Faculdade de Medicina, Programa de Pós-Graduação em Saúde Pública, Fortaleza, CE, Brasil.; 2 Universidade de Fortaleza, Centro de Ciências da Saúde, Fortaleza, CE, Brasil.; 3 Secretaria da Saúde do Estado do Ceará, Fortaleza, CE, Brasil.

**Keywords:** Rabies, Zoonosis, Epidemiology, Public Health

## Abstract

**INTRODUCTION:**

Rabies is considered one of the most relevant public health problems owing to its high fatality rate and the high number of deaths worldwide.

**METHODS:**

We included patients with human rabies who attended a reference hospital in the state of Ceará during 1976-2019.

**RESULTS:**

Data were available for 63 out of 171 (36.8%) patients. Of these patients, 48 (76.2%) were attacked by dogs. In recent years, wild animals have been the most common aggressor species (marmosets and bats). Only 39 (70%) patients were initially correctly suspected with rabies. Bites were the most frequent exposure (56; 96%), most commonly on the hands (21; 42%) and the head (9; 18.4%). Only 14 (22%) patients had sought medical assistance before the onset of symptoms, and only one completed post-exposure prophylaxis. The most prevalent signs and symptoms included aggressiveness/irritability (50; 79.4%), fever (42; 66.7%), sore throat/dysphagia (40; 63.5%), and myalgia (28; 44.4%). Hydrophobia was present in 17 patients (22.0%).

**CONCLUSIONS:**

Most cases of human rabies in Ceará occurred due to the failure to seek medical assistance and/or the failure of the health system in initiating early post-exposure prophylaxis. There is a need for specific information and education campaigns focusing on the cycle of sylvatic rabies as well as prevention measures. Health professionals should undergo refresher training courses on the signs and symptoms of rabies and on the specific epidemiological features of the disease in Brazil.

## INTRODUCTION

Human rabies (HR) is a zoonotic viral disease that affects the nervous system. It is a vaccine-preventable disease; however, its course is practically irreversible after the onset of signs and symptoms^1^. The disease causes approximately 60,000 human deaths per year worldwide, with the highest number of cases recorded in rural areas in Africa and Asia^2^ ^,^ ^3^. The main transmitter is the domestic dog (*Canis familiaris*), which accounts for approximately 99% of cases worldwide^1^.

In Brazil, a significant reduction in mortality rates due to HR has been achieved in the last 30 years after the implementation of canine vaccination campaigns and the intensification of the provision of post-exposure prophylaxis. Currently, there are only sporadic cases of HR transmitted by dogs^4^. In the last decade, 9 (23.7%) of the 38 HR cases registered in Brazil were transmitted by dogs. The other cases were primarily transmitted by hematophagous bats (52.6%), followed by non-human primates (10.5%) and felines (10.5%)^4^. In fact, the hematophagous bat (*Desmodus rotundus*) is the main transmitter of rabies virus to humans in Brazil, and the variant maintained and transmitted by this species is commonly found in human cases where other species, mainly dogs and cats, are aggressors^5^ ^,^ ^6^.

In the state of Ceará in Northeast Brazil, dogs, hematophagous bats (*Desmodus rotundus*), and other wild species are responsible for maintaining and transmitting variants of the rabies virus to humans^5^ ^-^ ^7^. Of the 46 HR cases registered during 1990-2016 in the state, 52% occurred in Fortaleza, and dogs were the most common transmitters of the virus. The other cases were distributed throughout the state's municipalities, and the virus was transmitted by marmosets (*Callithrix jacchus*) (26%), bats (8.7%), and raccoons (2.2%)^7^ ^-^ ^10^. In fact, since 2004, wild animals, especially marmosets, have become more prominent in the transmission chain in Ceará^7^ ^-^ ^9^. Recent studies have shown that bats are the main reservoirs for the circulation of the HR virus in Ceará^8^. The last case of HR in the state occurred in 2016; the virus was transmitted through a hematophagous bat bite on a farmer who did not seek medical assistance^11^.

Post-exposure prophylaxis after aggression by a rabid animal is highly effective and prevents disease and death. Early diagnosis and the initiation of post-exposure prophylaxis are essential. However, physicians generally have limited knowledge about signs, symptoms, and prophylaxis^12^. Owing to the complexity of the clinical signs, its sporadic occurrence, and the lack of experience of physicians in treating rabies patients, the diagnosis may be delayed. 

This study describes the clinical and epidemiological characteristics of 63 patients affected by human rabies in the state of Ceará over a period of 44 years. 

## METHODS

**FIGURE 1:**
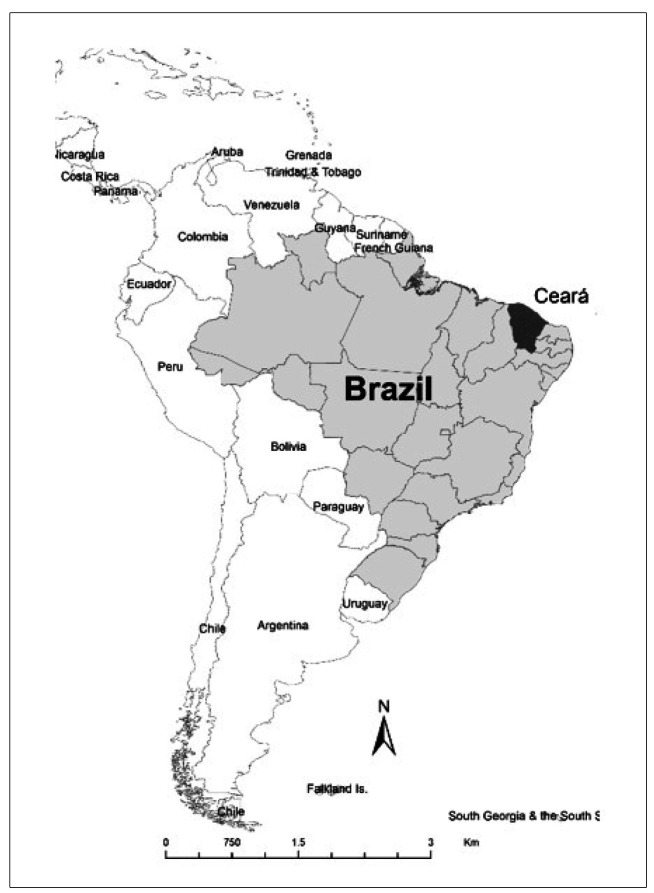
Location of the State of Ceará in Northeast Brazil in Latin America.

The study was carried out in the state of Ceará in Northeast Brazil (population: 8.8 million, Figure 1). In the state, 171 human rabies cases were reported during 1970-2019, with a constant decrease in the number of cases since 1978, after the start of intensive animal vaccination campaigns^7^. The last human rabies case transmitted by a dog occurred in 2010 and that transmitted by a sylvatic animal (hematophagous bat, *Desmodus rotundus*) occurred in 2016. 

The target population included all human rabies cases from which data were available in records, that is, from 1976 until the last registered case in 2016, attended by the state reference hospital for infectious diseases in the state capital Fortaleza (São José Hospital of Infectious Diseases). All registered rabies cases were treated at this hospital, as this is the only reference hospital for infectious diseases in the state. The analysis included all patient charts encountered in the hospital archives.

The variables available and analyzed in this study included epidemiological and clinical data, such as aggressor species, type of aggression/exposure to the virus, location of lesion, post-exposure prophylaxis, origin of the animal, animal situation, follow-up on animal, animal observation, incubation period, signs and symptoms, initial diagnosis, aggression dates, onset of symptoms, and death.

Data entry, processing, and analysis were performed using Microsoft Excel 2010 software within a database. The virus incubation period was calculated as the difference in days between the aggression date and the date of symptom onset. The patients' lifetimes were calculated based on the difference in days between the date of death and the date of the onset of clinical signs.

The study was approved by the Ethical Review Boards of the Federal University of Ceará (CAAE number: 13466719.6.0000.5054), of the State Health Secretariat (CAAE number - 13466719.6.3001.5051), and of the Reference Hospital for Infectious Diseases São José (CAAE number: 13466719.6 .3002.5044).

## RESULTS

Sixty-three patient charts were included in the analysis, representing 36.8% of the 171 patients who attended the reference hospital during the study period. Table 1 shows the epidemiological characteristics of patients. Approximately 25% of cases involved dogs as aggressors, and bites were by far the most frequent type of exposure, mostly on the hands (Table 1). Only 14 (22%) patients sought medical services after the attack (before the onset of specific symptoms). No treatment was prescribed for two of these patients, and the remaining patients received different prescriptions for post-exposure prophylaxis with a variation of 1 to 13 doses of rabies vaccine. Of these patients, nine (75%) did not complete the treatment. The origin of the aggressor animal was available only in 33 (52.3%) medical records, with approximately one-third (36.4%) being stray dogs. 

**TABLE 1:** Characteristics of human rabies cases according to the aggressor animal species, exposure type, injury site, post-exposure prophylaxis, animal origin, animal condition, and animal monitoring (N = 63), Ceará, Brazil, 1976-2019.

Variable	Number	%
**Aggressor animal species**		
Dog	48	76.2
Marmoset	6	9.5
Hematophagous bat	5	7.9
Unknown	3	4.8
Cat	1	1.6
Exposure type*		
Bite	56	96.6
Scratch	2	3.4
Lesion location*		
Hand	21	42.9
Head	9	18.4
Upper limbs	5	10.2
Lower limbs	5	10.2
Foot	3	6.1
Trunk	3	6.1
Head + upper limb	2	4.1
Hand + upper limb	1	2.0
**Post-exposure prophylaxis**		
Did not seek assistance	49	77.8
13 doses of anti-rabies serum	3	4.8
1 dose of vaccine	3	4.8
No treatment recommendation	2	3.2
3 doses of vaccine	2	3.2
11 doses of vaccine	1	1.6
Completed treatment	1	1.6
10 doses of vaccine + antiserum + booster every 10 days	1	1.6
7 doses of vaccine	1	1.6
Animal origin*		
Stray dog	12	36.4
Domestic	11	33.3
Wild	10	30.3
Animal condition*		
Abnormal behavior	35	85.4
No symptoms at the time of aggression	5	12.2
Unknown	1	2.4
Follow-up on animal*		
Dead	26	63.4
Disappeared	11	26.8
Unknown	4	9.7

* Data not available for all cases (see Materials and Methods).

The most prevalent signs and symptoms included changes in behavior (aggressiveness/irritability), followed by fever and pain in the throat/dysphagia (Table 2). Hydrophobia was common. Therapy was based on antibiotics, antivirals, anticonvulsants, analgesics, and antipyretics. The four cases of human rabies that occurred since 2010 were submitted to the so-called Recife protocol, but none of them survived.

**TABLE 2:** Signs and symptoms of rabies patients (N = 63), Ceará, Brazil. 1976-2019.

Signs and symptoms	Number	%
Behavior change (aggressiveness/irritability)	50	79.4
Fever	42	66.7
Sore throat/Dysphagia	40	63.5
Myalgia	28	44.4
Paresthesia	22	34.9
Hydrophobia	17	27.0
Headache	17	27.0
Sensitivity to drafts	16	25.4
Tremor	15	23.8
Insomnia	14	22.2
Inability to walk	14	22.2
Anorexia	13	20.6
Nausea/Vomiting	11	17.5
Prostration	11	17.5
Shortness of breath	11	17.5
Dysarthria (speech disorder)	10	15.9
Sweating	7	11.1
Hoarse cough	6	9.5
Seizures	6	9.5
Photophobia	3	4.8
Difficulty in urination and evacuation	2	3.2

Only 39 (70%) of the patients had an initial diagnosis of rabies when they sought the health unit after presenting the clinical signs of the disease; the other cases were first diagnosed as acute encephalitis (4; 7.1%) and meningitis (3; 5.4%), followed by a single diagnosis of respiratory infection, poliomyelitis, acute abdomen, acute polyencephalitis, pneumonia, nervousness/pneumonia/meningitis/rabies, head trauma, intestinal obstruction, dengue/meningitis, and Guillain-Barré syndrome. 

The mean time of the incubation period, in days, for patients being attacked by dogs was 49.2 (amplitude: 13-149), by marmosets 44.8 (20-61), by hematophagous bats *Desmodus rotundus* 33.5 (14-68), and for the patient being bitten by a cat, it was 118 days.

**FIGURE 2:**
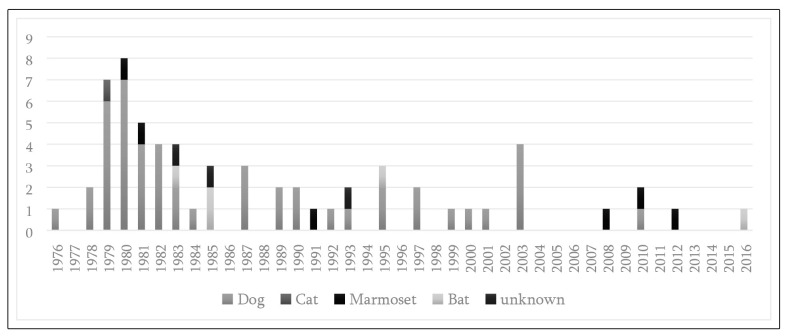
Human rabies cases by year of occurrence and aggressor animal species (N=63), Ceará, Brazil, during 1976-2016.

In general, dogs were the main transmitters, with six and seven cases in 1979 and 1980, followed by six cases in 1982 and 2003, respectively. Six cases transmitted by marmosets were recorded in 1980, 1981, 1991, 2008, 2010, and 2012. Of the five cases transmitted by bats, two occurred in 1985, and single cases occurred in 1983, 1995, and 2016. A single case transmitted by a cat occurred in 1979 (Figure 2).

The mean lifespan of patients after being attacked by dogs and cats was 5.8 and 3.0 days, respectively. After the attack by marmosets and bats, the mean lifespan was 9.7 and 9.4 days, respectively.

## DISCUSSION

This is the first systematic study on the epidemiological and clinical characteristics of human rabies in the state of Ceará, covering a 44-year period. In the first few years, dogs were the main transmitters of the virus and were responsible for 75% of all cases. In recent years, wild animals have become the most important aggressor species. Most victims were not aware of the importance and necessity of seeking assistance after being attacked. Hands were the most common body parts affected through bites. Health professionals often failed to diagnose rabies and did not prescribe post-exposure prophylaxis over time.

The clinical signs presented were mostly related to behavioral changes, such as aggressiveness and irritability, but the initial diagnosis of the disease was dismissed in 30% of cases. This reinforces the importance of assessing any mammal exposure and suspecting human rabies whenever a patient is seen in the health care system with a sudden onset of neurological symptoms. During an outbreak of human rabies in the state of Amazonas in Brazil, most victims presented similar neurological disorders such as paraparesis (87.5%), aggression and/or agitation (37.5%), mental confusion (25%), and tetraparesis (18.8 %)^13^. In another study in Brazil, patients presented with fever (92.6%), agitation (85.2%), paresthesia (66.7%), and dysphagia/paralysis (51.9%)^14^.

A single patient was attacked by a cat with a longer incubation period, presenting a 118-day interval that does not match the rabies incubation period commonly observed in human beings, which is, on average, 45 days^15^. Nevertheless, the incubation period can vary from days to years, depending on the location, severity, and depth of the lesion and the distance from the wound site to the brain and nerve trunks, the amount of virus inoculated, and the viral strain^15^. In a human rabies outbreak in the state of Amazonas due to attacks by hematophagous bats, 15 patients presented an incubation period of 16 to 39 days, but in one case, it took 120 days until the manifestation of clinical signs^13^. In Brazil, human rabies cases reported from 2007 to 2017 had a median incubation time of 50 days^14^. All these periods are sufficient to seek medical assistance and to initiate postexpouse prophylaxis, preventing the development of the disease and further deaths.

The lifespan of patients counting from the onset of clinical signs until death was within the period of 10 days, consistent with the lifetime of a patient affected by rabies described in the Brazilian Health Surveillance Guide^15^. In the state of Amazonas during the outbreak of cases transmitted by bats, deaths occurred between two and nine days after the onset of symptoms^13^. 

Despite attempts to cure the last four patients since 2010 in the state of Ceará, none has had a successful response. The affected patients were mostly treated with antibiotics and antivirals. After the onset of clinical signs, the chances of survival are remote and evidence was restricted to a few case reports^16^. Treatment is based on the Milwaukee/Recife protocol and is recommended for any patients with clinical suspicion of rabies who have an epidemiological link and inadequate post-exposure prophylaxis. The patients must be promptly isolated and admitted to the intensive care unit, providing central venous access, delayed bladder catheterization, and nasoenteral catheterization^12^. Of the 93 patients who received the Milwaukee protocol worldwide so far, 18 survived the disease. Countries with the largest number of survivors included India (five cases), the United States (three cases), and Brazil (two cases). The recovered patients had mild to severe sequelae, with the exception of three cases in the United States who had only mild sequelae^17^.

Dogs, especially stray dogs, are the most common aggressor species in many settings^14^ ^,^ ^18^ ^-^ ^20^. This is a consequence of the lack of public policies related to the control of stray dog populations and the fact that pet dogs have the closest relationship with humans. In fact, the control of stray dogs has been considered a priority for the prevention and control of human rabies^21^ ^,^ ^22^. At the beginning of the study period, the National Rabies Prophylaxis Program (*Programa Nacional de Profilaxia para a Raiva* - PNPR), based on mass vaccination campaigns of dogs and cats, an action that is considered of great importance for the control of the disease in the country, has not yet been established in the state of Ceará. Implementation started in 1973, with roll-out in all Brazilian states during the four years thereafter^23^. This explains the early period from 1979 to 1980, with the highest records of human rabies cases due to aggression by dogs found in our study. One third of the dogs that were attacked were stray dogs and had clinical characteristics typical of rabies. 

The cases involving wild mammals in this study have been reported since the 1980s. In particular, marmosets are responsible for transmitting the rabies virus to humans. In Ceará, it is common for the rural population to capture and raise non-human primates, such as pets, including marmosets. Because these rural populations are usually unaware of the rabies virus transmission cycle, they do not seek medical assistance after being attacked by a wild animal. 

In fact, the marmoset (*Callithrix jacchus*) has been the main transmitter of the rabies virus to humans in recent years in Ceará. It harbors a unique and exclusive variant, identified for the first time in Ceará, and thus far, this variant has been restricted to Northeast Brazil. Marmosets are the only terrestrial wild species responsible for human cases through direct aggression in the country^7^ ^,^ ^10^ ^,^ ^35^. Antigenic analysis and genetic sequencing of the rabies virus in wild and domestic animal species in Ceará allow a better understanding of the dynamics of disease transmission, to improve surveillance and control actions, consequently with the non-occurrence of new cases, serving as an example for Brazil and worldwide^5^ ^-^ ^9^.

The last record of a rabies case transmitted by a marmoset was in 2012 in a rural setting. The state rabies control program subsequently implemented measures to reduce the risk of rabies transmitted by wild animals, by actions such as the provision of information and education campaigns, and training of health professionals on all levels. 

By contrast, in the United States, bats were responsible for 70% of 125 human rabies cases recorded from 1960 to 2018^24^. Cases transmitted by hematophagous bats have also appeared in rural Ceará since 1985. In three cases that occurred in 1983, 1985, and 1993, it was not possible to identify the aggressor animal. Probably, the animal involved was the hematophagous bat *Desmodus rotundus*. It can happen that the victims are used as a food source for these animals and, due to the size of the wound and the subtlety of the animals’ attack, they often go unnoticed^25^. However, it was not possible to confirm the type of viral variant present, as we do not yet have evidence for this in Brazil. From 1996 to 2001, the identification of the rabies viral variant was carried out only for research purposes, and since 2002, antigenic identification with monoclonal antibodies was implemented at the Pasteur Institute in São Paulo^36^. 

Only one case was transmitted by a cat in 1979, in Fortaleza, the capital city of Ceará. To date, there have been no records of rabies cases with cats as transmitters in the state's database. Although canine rabies is under control in Ceará, surveillance in domestic species still requires attention, as treatment for disease transmitted through dogs accounts for more than 70% of post-exposure treatments in the state, after attacking humans^26^ ^,^ ^27^. Another concerning issue is the maintenance of the rabies virus by wild animals such as bats, crab-eating fox (*Cerdocyon thous*), marmosets (*Callithrix jacchus*), and raccoons (*Procyon cancrivorus*), as confirmed by the state laboratory^26^, which pose an imminent risk of transfer from wild to domestic species and secondary transmission to humans (bat-cat/dog-human)^28^. In Brazil, no immunization strategy is available against rabies for wild animal species, and the program relies on passive surveillance through viral monitoring of wild mammals found dead on the highways^4^. 

Only 22% of victims had sought medical care after exposure. There is still a need for actions to alert the population of Ceará on the main aspects of rabies, especially on prevention, and the awareness that post-exposure prophylaxis is the only form of prevention after being attacked by a diseased animal^15^. 

In other settings, limited access to the health care system is another key factor. For example, in South Africa, the main reason for not initiating post-exposure prophylaxis was the lack of health professionals^18^. In Ceará, the professionals treated patients but did not prescribe a dose of vaccine, probably because they were unaware of the human anti-rabies treatment protocol available on the Ministry of Health website, and the established post-exposure prophylaxis guidelines^29^. According to the human anti-rabies prophylaxis protocol of the Brazilian Ministry of Health, post-exposure prophylaxis is indicated after aggression by wild animals and domestic animals roaming freely, or without the possibility of observation^29^. Post-exposure prophylaxis is available free of charge within the nationwide Unified Health System (*Sistema Único de Saúde*). 

Another Brazilian study on 82 cases of human rabies transmitted by wild animals, mostly bats, reported that post-exposure prophylaxis was not prescribed for 72% of patients^30^. This is considered a major failure in health services because it is essential that professionals on the frontline of rabies surveillance have adequate knowledge and are aware of the risk of transmission, especially by wild animals. In 2020, in the northeastern Brazilian state of Paraíba, a woman was bitten on the hand by a wild canid, contracted rabies, and died. On the day she was attacked, she sought health services, but no recommendation for post-exposure prophylaxis was given by the health professional^31^. Human cases still occur and are related to the lack of awareness of the population followed by inadequate healthcare-seeking behavior, and by the failure of the health care system, leading to missed opportunities to save lives^14^ ^,^ ^34^. There is a clear need to update professionals working in rabies surveillance and treatment. 

Several patients abandoned post-exposure prophylaxis, compromising the body’s immune response and favoring disease development. In Mozambique in 2014, similar observations were made, and many patients did not receive full treatment to develop the disease^19^. In other countries, such as India, a minority of rabies victims received complete post-exposure prophylaxis, mainly due to the absence of vaccine and antiserum^19^ ^,^ ^20^ ^,^ ^32^. 

Even though canine rabies is controlled in Ceará, there is a high risk that dogs and cats might be contaminated by wild animals and then the virus transmission may restart to humans. To control rabies maintained and transmitted by dogs and humans with virus transmission by wild species, the state of Ceará performs surveillance actions in an integrated and continuous manner, in collaboration with different institutions. 

The state develops strategies that consider the local situation and conditions, providing the availability of supplies in a timely manner to ensure a better response and the non-occurrence of human cases. 

To increase effectiveness, rabies control requires a multidisciplinary, one-health approach. The disease is associated with all four determinant groups described by One Health as causing disease^33^: First, as a zoonosis, animal health plays an important role; second, populational and social factors such as poverty, inadequate living conditions, access to education, and human behavior are interlinked with the disease; third, the health care system, surveillance, professional training, and access to health care are pivotal factors; and finally, environment and climate change can be considered as additional driving factors for the dynamics of virus transmission. As shown by our group previously and exemplified by the last 6 cases that occurred in Ceará^34^, intensified operational and implementation research efforts at this last mile are needed to identify bottlenecks in the control programs that are specific for each setting.

This study had several limitations. We analyzed the secondary data, and some medical charts did not provide complete information. In addition, it was possible to include only 37% of registered rabies cases during the study period because the other charts could not be encountered in the archive. The information contained in the oldest medical records, such as data of type of exposure, lesion location, initial diagnosis, and origin of the attacking animal, was not complete. Despite these limitations, this study provides information of great relevance to human rabies. 

It can be concluded that most human rabies cases in Ceará occurred due to the failure to seek assistance and due to the failure of the health care system to initiate and complete early post-exposure prophylaxis. There is a need for specific information and education campaigns focusing on the sylvatic rabies cycle and prevention measures. Health professionals need refresher training courses on the signs and symptoms of rabies and on the specific epidemiologic features of the disease in Brazil.
